# Pathophysiological relationship between COVID-19 and olfactory dysfunction: A systematic review

**DOI:** 10.1016/j.bjorl.2021.04.001

**Published:** 2021-04-25

**Authors:** Mateus Henrique de Las Casas Lima, Ana Luiza Brusiquesi Cavalcante, Sydney Correia Leão

**Affiliations:** Universidade Federal do Vale São Francisco (UNIVASF), Paulo Afonso, BA, Brazil

**Keywords:** SARS-CoV-2, Anosmia, COVID-19, Olfaction disorders, Smell

## Abstract

**Introduction:**

SARS-CoV-2 is the pathogen of COVID-19. The virus is composed of the spike, membrane and envelope. On physiological smell, odoriferous substances bind to proteins secreted by sustentacular cells in order to be processed by olfactory receptor neurons. Olfactory disorder is one of the main manifestations of COVID-19, however, research is still required to clarify the mechanism involved in SARS-CoV-2 induced anosmia.

**Objective:**

This article aims to analyze current scientific evidence intended to elucidate the pathophysiological relationship between COVID-19 and the cause of olfactory disorders.

**Methods:**

Pubmed, Embase, Scopus and ScienceDirect were used to compose this article. The research was conducted on November 24th, 2020. Original articles with experimental studies in human, animal and in vitro, short communications, viewpoint, published in the English language and between 2019 and 2020 were included, all related to the pathophysiological relationship between olfactory disorders and COVID-19 infection.

**Results:**

Both human cell receptors ACE2 and TMPRSS2 are essential for the SARS-CoV-2 entrance. These receptors are mostly present in the olfactory epithelium cells, therefore, the main hypothesis is that anosmia is caused due to damage to non-neuronal cells which, thereafter, affects the normal olfactory metabolism. Furthermore, magnetic resonance imaging studies exhibit a relationship between a reduction on the neuronal epithelium and the olfactory bulb atrophy. Damage to non-neuronal cells explains the average recovery lasting a few weeks. This injury can be exacerbated by an aggressive immune response, which leads to damage to neuronal cells and stem cells inducing a persistent anosmia. Conductive anosmia is not sufficient to explain most cases of COVID-19 induced anosmia.

**Conclusion:**

Olfactory disorders such as anosmia and hyposmia can be caused by COVID-19, the main mechanism is associated with olfactory epithelium damage, targeting predominantly non-neuronal cells. However, neuronal cells can also be affected, worsening the condition of olfactory loss.

## Introduction

SARS-CoV-2 surfaced in late 2019 in the province of Wuhan, China. Soon after, the virus disseminated throughout the world, becoming pandemic. SARS-CoV-2 contains as genetic material a simple strain RNA and is composed mainly of three protein structures: the spike, the envelope, and the membrane. The spike binds to the Angiotensin-Converting Enzyme 2 receptor (ACE2) and both the envelope and the membrane involves the genetic material.^1^

Even though SARS-CoV and SARS-CoV-2 use the same entry path, there are two crucial differences in SARS-CoV-2 binding domain which allow a higher affinity to the host ACE2 receptor and, as a consequence, enhance its virulence. One difference lies on the alteration of two viral hotspots, hotspot 31 and hotspot 353. These salt bridges became more stabilized in the binding site of SARS-CoV-2. The second difference is a structural change in the ACE2 binding ridge caused by a four-residue motif, resulting in a more compact and stronger contact.^2^

In physiological smell, the molecules are detected by olfactory receptor neurons (ORN), which are bipolar neurons with a thin dendrite that contains at its end a button with 10–20 cilia, present in the pseudostratified epithelium located in the nasal cavity.^3^ There are also non-neuronal cells, such as sustentacular cells and Bowman cells, responsible for secreting mucus for dissolving and detecting odorants. At the other end of the neuron, a thin unmyelinated axon joins other axons, forming several bundles that cross the submucosal lamina and connect to the olfactory bulb.^1^, ^3^, ^4^, ^5^

Odoriferous substances require binding to specific proteins released in the extracellular space by sustentacular cells in order to be processed by the olfactory neuronal receptors.^3^ Three main functions of these proteins are suggested by studies: introduction of odoriferous substances to olfactory receptors, cleavage of hydrophobic binders to the aqueous phase and promotion of odoriferous substances withdrawal from the olfactory receptors.^3^, ^5^ Receptor activation then leads to transduction cascades that produce action potentials within the olfactory receptor neurons.^3^, ^6^

Initially, olfactory disorders, like anosmia (total loss of smell) or hyposmia (partial loss of smell),^7^ were not considered relevant symptoms, however, some studies indicated a possible relationship between these disorders and COVID-19.^8^, ^9^ After a wide viral propagation in Europe, it soon became evident that anosmia and hyposmia were both important means of disease diagnosis.^10^, ^11^ Therefore, the World Health Organization (WHO) acknowledged olfactory dysfunctions as primary symptoms of COVID-19.

This article aims to analyze current scientific evidence intended to elucidate the pathophysiological relationship between COVID-19 and the cause of olfactory disorders.

## Methods

This systematic review has as its purpose the clarification of the following question: “What is the pathophysiological relationship between olfactory disorders and COVID-19 disease?”. This article was composed according to the Preferred Reporting Items for Systematic Reviews and Meta-Analyses Statement (PRISMA) checklist.

### Inclusion criteria

Original articles with experimental studies in human, animal and in vitro, short communications, viewpoints, published on English language and between 2019 and 2020 were included, all related to the pathophysiological relationship between olfactory disorders and COVID-19 infection.

### Exclusion criteria

Articles not related to olfactory dysfunction and SARS-CoV-2, absent of pathophysiological mechanism on olfactory function, reviews and mini reviews, case report, errata, news, book chapters, letters to the editor, commentary, editorials, duplicated, perspective, not peer reviewed, not accessible, published in a non-English language and before 2019 were not included.

### Search strategy and study selection

Pubmed, Scopus, Embase and ScienceDirect were used. On Pubmed, MeSH indexed terms were used as well as Emtree indexed terms on the Embase platform. On ScienceDirect and Scopus free terms were used. All articles were gathered on 24th November 2020 using the following search strategies:

Pubmed: ((((“COVID-19”[Supplementary Concept]) OR (“coronavirus”[MeSH Terms])) AND ((“smell”[MeSH Terms]) OR (“olfaction disorders”[MeSH Terms])) NOT review[Filter] NOT systematic review[Filter] NOT editorial[Filter] NOT letter[Filter] NOT review[Title])) AND 2019:2021[dp]

Science Direct: (SARS-CoV-2 OR covid-19) AND (“olfactory disorder” OR “smelling disorder” OR “olfactory dysfunction” OR “loss of smell”)

Year: 2019−2021

Title: NOT review

Article types: Research articles; Conference abstracts; Data articles; Replication studies; short communications; other.

Embase: (‘coronavirus disease 2019’/exp/mj) AND (‘smelling disorder’/exp/mj) NOT (‘review’:ti OR ‘editorial’:it OR ‘review’:it OR ‘erratum’:it OR ‘letter’:it OR ‘note’:it OR ‘case report’:it OR ‘case study’:it OR ‘systematic review’:it OR ‘interview’:it) AND [2019–2021]/py

Scopus: (TITLE-ABS-KEY(COVID-19 OR SARS-CoV-2) AND TITLE-ABS-KEY(smelling AND disorder)) AND PUBYEAR > 2018 AND NOT DOCTYPE (bk OR ch OR bz OR cr OR ed OR er OR le OR mm OR no OR pr OR rp OR tb OR re OR sh) AND NOT TITLE(review)

All citations were managed using Zotero in order to eliminate duplicates and organize the article selection steps consistent with inclusion and exclusion criteria. The titles and abstracts were analyzed by two independent reviewers, discarding articles not compatible with the inclusion criteria. Consecutively, the texts were read fully and again evaluated by both reviewers independently. Divergences were discussed to reach a consensus. The quality assessment was written in a descriptive and unbiased manner. There was no blind review of the authors and institutions of the studies under review.

### Data extraction and analysis

The data analysis was performed descriptively. The data was extracted individually by the reviewers using standardized key questions associated with the pathophysiological relationship between olfactory disorders and COVID-19 infection as well as olfactory epithelial damage mechanisms and the duration of these disorders.

## Results

**Figure 1:**
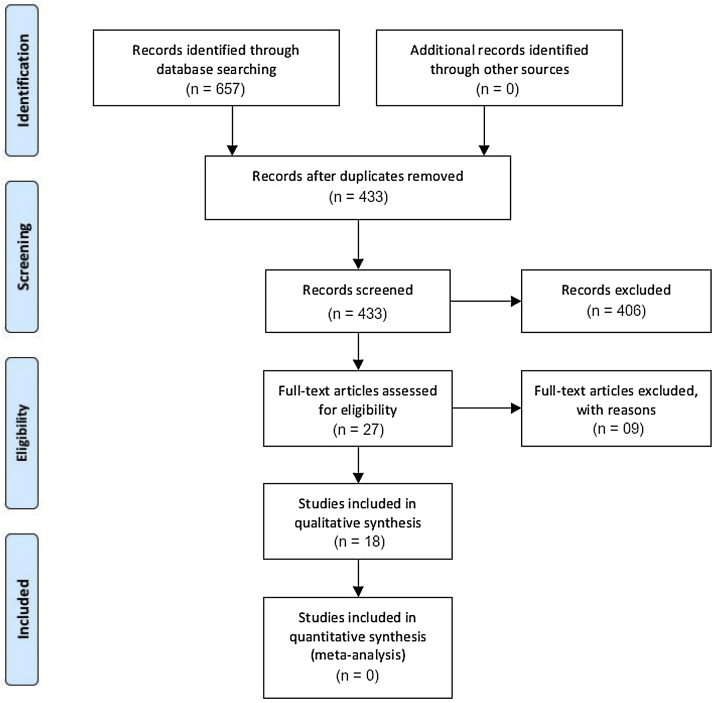
Flowchart according to PRISMA protocol.

In total 657 articles were extracted, 148 on Scopus, 190 on Embase, 167 on PubMed and 152 on ScienceDirect. After duplicates were removed using Zotero, 433 articles remained. Sequentially, after title and abstract were evaluated according to eligibility criteria, 27 articles remained. Lastly, after full text assessment, there were 18 articles left (Fig. 1 and Table 1, Table 2).

**Table 1:** Excluded articles table.

Title	Author	Location	Year	Reason for exclusion
Acute-onset smell and taste disorders in the context of COVID-19: a pilot multicentre polymerase chain reaction-based case-control study	Beltrán-Corbellini et al.	Spain	2020	Absent of relevant pathophysiological mechanisms
Autonomic Brain Centers and Pathophysiology of COVID-19.	Chigr et al.	Morocco	2020	Absent of relevant pathophysiological mechanisms
Clinical and Radiological Evaluations of COVID-19 Patients with Anosmia: Preliminary Report	Lechien et al.	Belgium	2020	Absent of relevant pathophysiological mechanisms
Common determinants of severe Covid-19 infection are explicable by SARS-CoV-2 secreted glycoprotein interaction with the CD33-related Siglecs, Siglec-3 and Siglec-5/14	Murch et al.	United Kingdom	2020	Absent of relevant pathophysiological mechanisms
COVID-19 treatments and pathogenesis including anosmia in K18-hACE2 mice	Zheng et al.	USA	2020	Absent of relevant pathophysiological mechanisms
COVID-19 viral load in the severity of and recovery from olfactory and gustatory dysfunction	Cho et al.	Hong Kong	2020	Absent of relevant pathophysiological mechanisms
Gaining back what is lost: recovering the sense of smell in mild to moderate patients after COVID-19	Ianuzzi et al.	Italy	2020	Absent of relevant pathophysiological mechanisms
Inhibition of focal adhesion kinase increases adult olfactory stem cell self-renewal and neuroregeneration through ciliary neurotrophic factor	Jia et al.	USA	2020	Absent of relevant pathophysiological mechanisms
Olfactory dysfunction during COVID-19 pandemic	Izquierdo-Domínguez et al.	Spain	2020	Absent of relevant pathophysiological mechanisms

**Table 2:** Included articles table.

Title	Author	Location	Year	Study design	Information’s collected
A single-cell RNA expression map of human coronavirus entry factors	Singh et al.	Germany	2020	Original research	Expression of TMPRSS4 as an alternative protein
Acute onset olfactory/taste disorders are associated with a high viral burden in mild or asymptomatic SARS-CoV-2 infections	Nakagawara et al.	Japan	2020	Short communication	Enhanced expression of ACE2 and TMPRSS2 in the nasal epithelia
Anosmia in COVID-19 associated with injury to the olfactory bulbs evident on MRI	Aragão et al	Brazil	2020	Research	Transmission of SARS-CoV-2 through the olfactory nerve and injury to the olfactory bulb
Anosmia in COVID-19: A bumpy road to establishing a cellular mechanism	Bilinska et al.	Poland	2020	Viewpoint	Mechanism of non-neuronal cells injury
Anosmia in COVID-19: underlying mechanisms and assessment of an olfactory route to brain infection	Butowt et al.	Poland	2020	Research	Relationship between the immune response and anosmia worsening
Cerebral micro-structural changes in COVID-19 patients – an MRI-based 3-month follow-up study	Lu et al.	China	2020	Research	Transmission of SARS-CoV-2 through the olfactory nerve and damage to the olfactory bulb
Co-expression of peripheral olfactory receptors with SARS-CoV-2 infection mediators: Potential implications beyond loss of smell as a COVID-19 symptom	Kerslake et al.	United Kingdom	2020	Research	Mechanism of SARS-COV-2 endocytosis and expression of ACE2 and TMPRSS2 in non-neuronal cells
Frequency and outcome of olfactory impairment and sinonasal involvement in hospitalized patients with COVID-19	Jalessi et al.	Iran	2020	Prospective study	Conductive anosmia as an unlikely mechanism in SARS-CoV-2 infection
Loss of smell in COVID-19 patients: MRI data reveals a transient edema of the olfactory clefts	Eliezer et al.	France	2020	Prospective study	Anosmia worsening caused by damage to the olfactory bulb
Massive transient damage of the olfactory epithelium associated with infection of sustentacular cells by SARS-CoV-2 in golden Syrian hamsters	Bryche et al.	France	2020	Research	Mechanism of olfactory epithelium injury and regeneration time
Neurological insights of COVID-19 pandemic.	Das et al.	India	2020	Viewpoint	Higher binding affinity of SARS-CoV-2 to ACE2, compared to the MERS-Cov
Non-neuronal expression of SARS-CoV-2 entry genes in the olfactory system suggests mechanisms underlying COVID-19-associated anosmia	Brann et al.	USA and United Kingdom	2020	Research	Expression of TMPRSS4 as an alternative protein and mechanism of SARS-COV-2 endocytosis
Objective evaluation of the nasal mucosal secretion in COVID-19 patients with anosmia	Islamoglu et al.	Turkey	2020	Original research	Conductive anosmia as an unlikely mechanism in SARS-CoV-2 infection
Olfactory bulb MRI and paranasal sinus CT findings in persistent COVID-19 anosmia	Kandemirli et al.	Turkey	2020	Original research	Anosmia worsening caused by damage to the olfactory bulb
Potential mechanisms for COVID-19 induced anosmia and dysgeusia	Eshraghi et al.	USA and United KIngdom	2020	Viewpoint	Conductive anosmia as an unlikely mechanism in SARS-CoV-2 infection and damage mechanism to non-neuronal cells
SARS-CoV-2: olfaction, brain infection, and the urgent need for clinical samples allowing earlier virus detection.	Butowt et al.	Poland	2020	Viewpoint	Nasal mucosa as the main route of entry of SARS-CoV-2 into the body and the relationship between the immune response and anosmia worsening
Taste and smell disorders in COVID-19 patients: Role of interleukin-6	Cazolla et al.	Italy	2020	Research	Relationship between the immune response and anosmia worsening
The cellular basis of the loss of smell in 2019-nCoV-infected individuals	Gupta et al.	India	2020	Viewpoint	Mechanism of SARS-COV-2 endocytosis and expression of ACE2 and TMPRSS2 in non-neuronal cells

Of the 18 selected studies, 9 were carried out in Europe, 7 in Asia, 1 in the United States and 1 in Brazil. Most of them were original research, from the mechanism of entry of the SARS-CoV-2 virus in the host to the pathophysiological mechanisms that cause anosmia.

### SARS-CoV-2 entry mechanism in the host organism

SARS-CoV-2 uses its spike protein to bind to ACE2, and this link is formed with the aid of transmembrane serine protease 2 (TMPRSS2). TMPRSS2 is a protease present on the surface of the target cell, which plays an important role in the virus entry pathway, as it cleaves a specific point of the spike protein, thus allowing a connection between the C-terminal domain (CTD) of pico protein and ACE2.^1^, ^5^, ^12^ Recent studies have shown that another transmembrane protease, TMPRSS4, is able to perform the same function as TMPRSS2, therefore being an alternative protease for SARS-CoV-2.^5^, ^13^ In addition to transmembrane proteases, there is also the intracellular protease known as Cathepsin-L, which can also be responsible for the entry of the virus.^12^

Compared to SARS-CoV, beside the greater stability of hotspots, SARS-CoV-2 CTD also has more van der Waals bonds, hence it binds with greater affinity to ACE2.^2^ Some tissues express ACE2, such as lungs, heart, oral and nasal mucosa rinses, testicles, intestines, lymphoid organs and brain, as a result, they are more susceptible to the invasion of SARS-CoV-2.^1^, ^5^, ^14^

**Figure 2:**
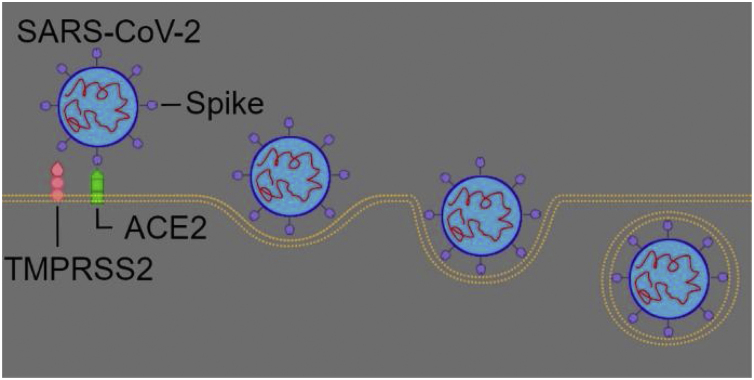
Mechanism of virus entry into the target cell using the ACE2 receptor aided by TMPRSS2 protease.

Nevertheless, the main entry route inside the organism is through the nasal mucosa (Fig. 2).^2^, ^14^, ^15^

### Possible mechanism of anosmia

**Figure 3:**
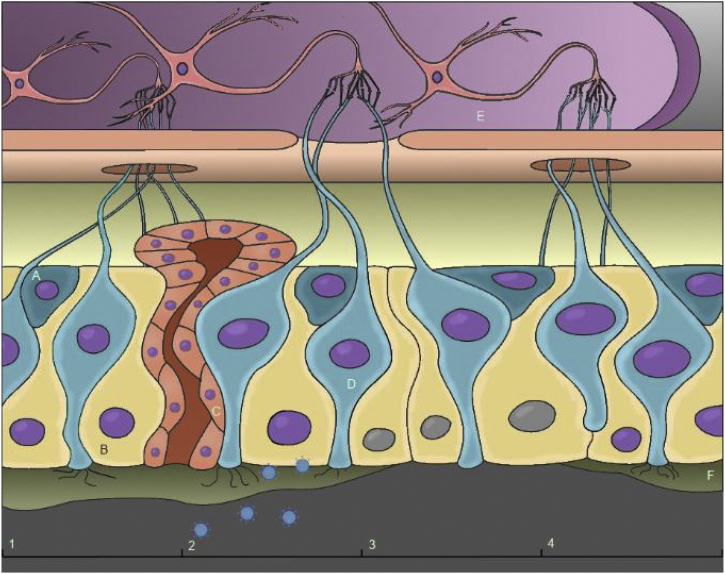
A, Basal stem cells; B, Sustentacular cells; C, Bowman cell; D, Olfactory receptor neurons; E, Olfactory bulb; F, Mucus. (1) Intact olfactory epithelium; (2) Arrival and invasion of SARS-CoV-2; (3) Damage to sustentacular cells, loss of cilia from olfactory receptor neurons and interruption of mucus production; (4) Regeneration of the olfactory epithelium.

Conductive anosmia occurs due to nasal obstruction, which is common in many viruses, and may be accompanied by rhinorrhea and rhinitis symptoms. Studies suggest however that the loss of smell in COVID-19 occurs, in most cases, regardless of these symptoms.^16^, ^17^, ^18^ Thus, this hypothesis, in the case of SARS-CoV-2, can be ruled out as the main mechanism causing anosmia. Injury to the olfactory epithelium is the mechanism identified as the most likely cause of olfactory disorders caused by SARS-CoV-2, which can be aggravated by damage to the central nervous system (Fig. 3).^16^, ^19^, ^20^

### Injury to the olfactory epithelium

Analyzes based on RNA sequencing showed considerable expression of ACE2 and TMPRSS2 in Sustentacular cells (SUS), Bowman cells and a small fraction in stem cells. In contrast, the presence of ACE2 in olfactory receptor neurons has not been confirmed.^5^, ^6^, ^12^, ^16^^,^^19^

It is known that the virus invades cells through ACE2 in conjunction with TMPRSS2, that said, SARS-CoV-2 has as its main target non-neuronal cells. Moreover, the average recovery of smell is 2 weeks, a time span not compatible with the regeneration of neuronal cells, substantiates this hypothesis.^16^, ^19^

In experiments with hamsters, infected with SARS-CoV-2 via nasal instillation, massive damage to the olfactory epithelium was found, only two days after infection. On the fourth day after infection, most of the epithelium had disappeared. After fourteen days, the epithelium showed signs of recovery, but it had not yet returned to normal. It was verified that regions where the damage was more intense, the axons of the olfactory receptor neurons were practically in contact with the external environment. The main observations in this experiment were: the infection and desquamation of the olfactory epithelium, the preference for the virus for sustentacular cells rather than neuronal cells and the intense recruitment of immune cells.^19^

Damage to sustentacular cells and Bowman cells directly affects the perception of odors, not by transmission of the virus to olfactory receptor neurons (NRO), but by impairing some of its functions that are necessary for the functional metabolism of these neurons.^1^, ^5^ Damage to Bowman cells would cause an interruption in the production of nasal mucus, necessary for the dissolution of odorous particles. Moreover, damage to sustentacular cells would result in a suppression of the removal of volatile products, through the cytochrome P450 route, a halt in the endocytosis of protein complexes that bind to odorants, after the transduction of signals to the NROs and interruption of the supply of additional glucose to the cilia of the NRO and a electrolyte and water imbalance.^5^ For this reason, damage to sustentacular cells would certainly influence odor perception, characteristic of anosmia and hyposmia. Furthermore, the infection of the sustentacular cells also generates a loss of the cilia of the olfactory receptors, which is illustrated in the impossibility of transmitting the odorous stimulus and, thus, detecting smells.^6^ MRI studies exhibit a correlation between bulb size and olfactory dysfunction, reflecting a lower sensory activity in the olfactory epithelium, which leads to less synaptogenesis in the olfactory bulb, decreasing its volume. This reduction in olfactory epithelial activity is a result of damage to non-neuronal cells, further corroborating this hypothesis.^16^, ^19^ The re-establishment of normosmia would be due to the rapid regeneration of sustaining cells from stem cells.^6^

Olfactory epithelium damage can be aggravated by an inflammatory response, leading to cell death, known as pyroptosis. The immune system is activated after pathogen recognition, causing an increase in the secretion of pro-inflammatory cytokines and chemokines: Interleukin-6 (IL-6), Interferon gamma (IFN-γ), chemoattractive proteins from monocyte chemoattractant protein-1 (MCP-1) and interferon-inducible protein 10 (IP-10). These cytokines are indicative of a reaction more focused on the recruitment of monocytes and T-lymphocytes.^16^, ^20^, ^21^ In addition, a study demonstrated a possible correlation between anosmia and IL-6 levels. IL-6 induces the expression of several acute-phase proteins, among them C-reactive protein, serum amyloid A, α1-antiquimotripsin, haptoglobin, fibrinogen and complement components. Therefore, patients with higher levels of IL-6 may be associated with more intense cases of olfactory disorders.^20^ The high production of cytokines can provoke olfactory neurons death. The olfactory epithelial neurons replacement by basal stem cells requires a longer recovery time, thus explaining persistent anosmia cases.^16^, ^20^, ^22^

Loss of smell may be due to olfactory bulb inflammation triggered by virus infection.^16^, ^19^, ^23^, ^24^ SARS-CoV has the ability to infect the central nervous system through the synapses, using the olfactory nerve afferents to reach the olfactory bulb, raising the possibility of SARS-CoV-2 utilizing this infection path as well.^21^, ^22^, ^25^

## Discussion

Epidemiological factors such as age and lifestyle directly interfere with the prevalence of olfactory disorders. Studies have presented a correlation between ACE2 receptor expression and age, with ACE2 being more prevalent in adults than children,^4^, ^26^ in agreement with this information, young people usually exhibit a less severe prognosis.^27^, ^28^, ^29^, ^30^, ^31^

Regarding life habits, it was found that smokers have a greater chance of developing olfactory disorders due to COVID-19, that is a consequence of a higher expression of the ACE2 receptor stimulated by the nicotinic acetylcholine receptor (nAChR).^2^ Another set of evidence indicated that women are more likely to develop olfactory disorders than men.^30^, ^31^, ^32^, ^33^ A possible explanation would be that incomplete X chromosome inactivation would contribute to increased expression of ACE2.^34^

Beside life habits and age, SARS-CoV-2 and population genetic mutations also contribute to a different prevalence of anosmia. At the beginning of the pandemic, the most common strain of SARS-CoV-2 was D614, which means that the virus’s spike protein has the amino acid D (aspartic acid) at position 614. Throughout the evolution of the pandemic, the amino acid glycine came to occupy position 614, giving rise to the G614 variant, which is considered the dominant one on the European continent. In East Asia, the dominant variant still is D614. Comparing both variants, it was found that D614 was slightly correlated with chemosensory disorders, such as anosmia, whereas G614 was highly correlated, consistent with the prevalence of anosmia in the European population found to be three times higher compared to the East Asian population.

Studies have demonstrated that the substitution of amino acids at position 614, from aspartic acid to glycine, favored the spike protein’s conformational opening state, resulting in an enhanced exposure of the binding domain of this protein and, as a result, increasing the probability of binding to ACE2. Due to this increased likelihood of binding to the host receptor, the G614 variant is directly associated not only with a higher incidence of anosmia, but also with a greater infectivity and transmissibility.^34^, ^35^

Moreover, studies have shown that there is a genetic variation in the human receptor for the virus, ACE2, in the European population, which increases the expression of this receptor. As a result, Europeans would be more predisposed to anosmia compared to Asians, precisely because they have more receptors for the entry of SARS-CoV-2.^36^, ^37^

## Conclusion

The SARS-CoV-2 entry pathway requires an ACE2 receptor, favored by the use of TMPRSS2 protease. Several organs express both ACE2 and TMPRSS2, including the olfactory epithelium. Olfactory disorders such as anosmia and hyposmia can be caused by COVID-19; the main mechanism is associated with olfactory epithelium damage, targeting predominantly non-neuronal cells. However, neuronal cells can also be affected, worsening the condition of olfactory loss.

## Conflict of interest

The authors declare no conflict of interest.
